# Ocular findings among patients surviving COVID-19

**DOI:** 10.1038/s41598-021-90482-2

**Published:** 2021-05-26

**Authors:** Ílen Ferreira Costa, Livia Pimenta Bonifácio, Fernando Bellissimo-Rodrigues, Eduardo Melani Rocha, Rodrigo Jorge, Valdes Roberto Bollela, Rosalia Antunes-Foschini

**Affiliations:** 1 Hospital das Clínicas, Ribeirão Preto Medical School, University of São Paulo, Ribeirão Preto, Brazil; 2 Department of Ophthalmology, Otorhinolaryngology, and Head and Neck Surgery, Ribeirão Preto Medical School, University of São Paulo, Ribeirão Preto, Brazil; 3 Department of Social Medicine, Ribeirão Preto Medical School, University of São Paulo, Ribeirão Preto, Brazil; 4 Department of Internal Medicine, Ribeirão Preto Medical School, University of São Paulo, Ribeirão Preto, Brazil

**Keywords:** Signs and symptoms, Eye manifestations

## Abstract

To describe the medium-term ophthalmological findings in patients recovering from COVID-19. Patients recovered from the acute phase of COVID-19 underwent a complete ophthalmological evaluation, including presenting and best-corrected visual acuity (BCVA), refractometry, biomicroscopy, tonometry, break-up time and Schirmer tests, indirect ophthalmoscopy, color fundus picture, and retinal architecture evaluation using optical coherence tomography. Socio-demographic data and personal medical history were also collected. According to the severity of systemic manifestations, patients were classified into mild-to-moderate, severe, and critical. Sixty-four patients (128 eyes) were evaluated 82 ± 36.4 days after the onset of COVID’s symptoms. The mean ± SD duration of hospitalization was 15.0 ± 10.7 days. Seven patients (10.9%) had mild-to-moderate, 33 (51.5%) severe, and 24 (37.5%) critical disease. Median [interquartile ranges (IQR)] presenting visual acuity was 0.1 (0–0.2) and BCVA 0 (0–0.1). Anterior segment biomicroscopy was unremarkable, except for dry eye disease, verified in 10.9% of them. The mean ± SD intraocular pressure (IOP) in critical group (14.16 ± 1.88 mmHg) was significantly higher than in severe group (12.51 ± 2.40 mmHg), both in the right (*p* 0.02) and left eyes (*p* 0.038). Among all, 15.6% had diabetic retinopathy, and two patients presented with discrete white-yellowish dots in the posterior pole, leading to hyporreflective changes at retinal pigment epithelium level, outer segment, and ellipsoid layers. The present study identified higher IOP among critical cases, when compared to severe cases, and discrete outer retina changes 80 days after COVID-19 infection. No sign of uveitis was found.

## Introduction

At the end of 2019, the rapid spread of a new coronavirus led to a severe acute respiratory syndrome (SARS-CoV-2), known as COVID-19, which was declared a pandemic in March 2020 by the World Health Organization^1^. Until now, it already killed more than 2.7 million people worldwide, including Dr. Li Wenliang, an ophthalmologist in Wuhan, Hubei, China, one of the first doctors to notice and warn for its severity and rapid spread^2^.

Among the patients with COVID-19 in the acute phase of the disease, approximately 10% exhibited ocular symptoms, particularly related to ocular surface (conjunctivitis, red eye, foreign body sensation, dry eye, photophobia, blurry vision, itching, epiphora, ocular pain, and floaters)^3–6^. Concerning posterior segment commitment, flame-shaped or microhemorrhages and cotton wool spots in the papillomacular bundle were shown^7–9^. Other reported systemic symptoms include shortness of breath, chest pain, headaches, neurocognitive difficulties, muscle pains and weakness, gastrointestinal upset, rashes, metabolic disruption (such as poor control of diabetes), thromboembolic conditions, depression, and other mental health conditions^10–12^.

Recently, the post-COVID syndrome (PCS) or post-COVID condition^13^ has been proposed, taking into account the high frequency (from 10 to 35%) of people affected by SARS-CoV-2 that persist with symptoms after the disease's acute phase^14^. Greenhalgh et al.^15^. defined PCS as extending beyond three weeks from the onset of first symptoms and chronic COVID-19 as extending beyond 12 weeks. The most common findings are cough, low-grade fever, and fatigue, all of which may relapse and remit. Other reported symptoms of the PCS also include shortness of breath, chest pain, headaches, neurocognitive difficulties, muscle pains and weakness, gastrointestinal upset, rashes, metabolic disruption, thromboembolic conditions, depression and other mental health conditions^16,17^. In these first publications about PCS, we did not find any information about ocular manifestations persisting after COVID-19, and it is not possible to know if they were not there or if they were not reported, investigated or documented.

The present study aimed to look for visual and ocular manifestations during the recovery phase as possible sequels of the disease.

## Methods

This study is nested within a large cohort study named RECOVIDA, aimed to comprehensively describe the clinical picture of the post-COVID-19 condition. Patients were recruited during follow-up by the infectious disease ambulatory care after the acute phase of the disease. Most of the patients attending this ambulatory have been previously hospitalized in Hospital das Clínicas de Ribeirão Preto complex with severe or critical clinical picture. A small proportion of patients presented mild-to-moderate disease and were not hospitalized during the disease’s acute phase.

We offered the opportunity to participate in this ophthalmologic cross-sectional study to patients attending the Post-Covid ambulatory care from July to November 2020. We included 64 patients (128 eyes). They were considered to be at the recovery phase of the disease when the time interval between the disease’s first symptoms and the eye examination was at least 30 days. Their diagnosis was based either on positive polymerase chain reaction for SARS-CoV-2 obtained on throat swab samples or nasopharyngeal specimen obtained before admission.

The patients were inquired about the ocular signs and symptoms in the recovery phase of the disease and also responded to a short questionnaire with three items: 1. How often do you feel your eyes dry? (0. Never, 1. Sometimes, 2. Often, 3. Constantly); 2. How often do you feel your eyes irritated? 3. Have you ever been diagnosed (by a clinician) as having dry eye syndrome? (1. Yes, 2. No). The response to the questionnaire was considered positive for dry eye when the responses for questions 1 and 2 were “Often” or “Constantly” or the response for question 3 was “Yes"^18,19^. They were also asked about the presence of blurry vision and ocular pain at the moment of the ophthalmologic examination and if these symptoms were previous to COVID-19 diagnosis or if they appeared simultaneously with COVID-19 and persisted until the exam day.

They were evaluated with a complete ophthalmological examination that included presenting and distant best-corrected visual acuity (DBCVA) displayed in logMAR. We also performed biomicroscopy and dry eye tests. The break-up time (BUT) assessment (BUT was considered positive for dry eye if < 7 s on the worse eye), corneal fluorescein staining (the corneal area was divided into five zones, one central area and four peripheric ones, each region was classified as no stain (= 0), 1, 2 or 3 (great stain), and a total score varying from 0 to 15 was calculated for the entire cornea, following the Dry eye workshop guidelines^20^. The exam was considered positive for dry eye if the corneal fluorescein staining score was ≥ 3 on the worse eye). The tear flow was measured by the Schirmer test without anesthesia and considered positive for dry eye if the worse eye showed ≤ 5 mm of wetness). Dry eye disease was defined when the positivity in the dry eye short questionnaire defined above was associated with positivity in at least one of the three dry eye tests mentioned above in at least one eye. Goldmann tonometry (mmHg) and refractometry with values displayed in spherical equivalent (sph eq), defined as spherical error plus half the cylindrical error, were also registered. Color fundus pictures were obtained using Topcon TRC-50DX and Nikon D90 cameras. Spectral domain optical coherence tomography (SD-OCT) was performed using the Heidelberg Spectralis HRA-OCT device (Heidelberg Engineering, Heidelberg, Germany). The macular architecture was evaluated using a standard 20° × 15° tracking protocol, consisting of 19 horizontal sections (each from 25 frames) with a distance of 240 μm between each scan, covering the 20° × 15° quadrilateral in the retina, centered on the fovea. To improve the accuracy of OCT data, the automatic delineation of the internal and external limits of the sensorineural retina, generated by the equipment's software, was verified for each of the scans.

All of the patients were also evaluated on a clinical basis and had an extensive data set concerning their systemic manifestations and severity of the disease, including previous comorbidities, body mass index (BMI), number of days of hospitalization, oxygen therapy, mechanical ventilation and sequels including neuromotor diseases. They were classified into *mild-to-moderate* (mild symptoms, no need for oxygen support or hospitalization); *severe* (severe symptoms, required hospitalization, most of them requiring oxygen support); and *critical* (severe symptoms, required hospitalization and intensive care, intubation and/or had specific complications)^14^.

Our local Ethics Committee (Comitê de Ética em Pesquisa do Hospital das Clínicas da Faculdade de Medicina de Ribeirão Preto) approved the RECOVIDA cohort and this ophthalmological cohort and we have followed the tenets of the Declaration of Helsinki. This prospective cross-sectional study obtained informed consent from all subjects.

All statistical analyses were performed using Stata (Stata/IC 15.1, College Station, TX). The Doornik-Hansen for multivariate normality test was used to look for Gaussian distribution. Categorical variables were analyzed using 2-sided Fisher’s exact test. Continuous variables were analyzed using the one-way ANOVA test, the Wilcoxon rank-sum (Mann–Whitney test), the Kruskal–Wallis test, and the Wilcoxon matched-pairs signed rank test. A *p* value of less than 0.05 was considered to be statistically significant. We looked for correlation between right and left eyes refractive errors using Pearson coefficient.

## Results

Concerning the demographics and clinical data related to the clinical manifestations of the disease, nine (14.0%) were healthcare professionals (one medical doctor, two registered nurses, and six auxiliary nurses), 29 (45.3%) were obese (body mass index higher than 30), 12 (18.7%) were previous smokers, and one is currently smoking (1.5%). Regarding previous comorbidities, 15 (23.4%) had no previous comorbidities, 19 (29.7%) had systemic arterial hypertension, 19 (29.7%) diabetes mellitus, and 12 (18.7%) patients had dyslipidemia. Among them, 46 (71.8%) used long-term medications.

Among all, 57 (89.0%) required hospitalization and oxygen support, 29 (45.3%) required intensive care, and 23 (35.9%) needed invasive mechanical ventilation. The most commonly used drugs were azithromycin in 29 (45.3%); heparin in 36 (56.2%); ceftriaxone in 33 (51.5%); and prednisone in 28 (43.7%). Regarding oxygen support interface type, the most frequently used was oxygen catheter / nasal cannula interfaces in 56 (87.5%); reservoir mask 15 (23.4%); and continuous positive airway pressure (CPAP) / noninvasive ventilation (NIV) in 11 patients (17.1%).

Based on clinical data, patients were classified into mild-to-moderate (7 patients, 10.9%); severe (33 patients, 51.5%); and critical (24 patients, 37.5%).

The mean ± SD duration of hospitalization was 15.0 ± 10.7 days. The mean ± SD interval (days) between the onset of COVID-19 symptoms and the day the ophthalmology team evaluated them was 82 ± 36.4 days.

Table 1 presents the demographic and ocular data of the 64 individuals. Presenting and DBCVA were significantly different (*p* ≤ 0.03, Wilcoxon matched-pairs signed-rank test) in mild-to-moderate, severe, and critical groups, except for the left eyes (LE) in the mild-to-moderate group. Only two eyes of two patients presented DBCVA > 0.5 logMAR due to cataract diagnosis. As the Pearson correlation for refractive errors between RE and LE was 0.85, we analyzed only the RE. Concerning refractive errors on the right eyes (RE), 20 (31.7%) had myopia < − 0.50D and 26 (41.2%) had hyperopia >  + 0.50D. The intraocular pressure (IOP) was statistically different when comparing severe and critical groups, both in RE (*p* = 0.022) and LE (*p* = 0.038). Also, the frequency of personal history of dry eye or severe symptoms was statistically different concerning the systemic severity of the disease and sex—the frequency was higher in mild-to-moderate cases (*p* = 0.011, two-tailed Fisher’s exact test) and in women (males: 4/33 (12.1%); females 12/31 (38.7%), *p* = 0.041, two-tailed Fisher’s exact test).

**Table 1:** Ocular and demographic data of patients recovered from COVID-19, according to the systemic classification of the disease severity (mild-to-moderate, severe, and critical).

	Mild-to-moderate (n = 7)	Severe (n = 33)	Critical (n = 24)	*p* value
Male/female	2:5	13:20	18:6	0.015
Age	46.7 (44.5–55.7)	55.6 (43.0–65.4)	53.9 (48.0–57.9)	0.509
Presenting VA RE	0.1 (0–0.2)	0 (0–0.2)	0.1 (0–0.2)	0.085
Presenting VA LE	0 (0–0)	0 (0–0.2)	0.1 (0–0.2)	0.585
DBCVA RE	0 (0–0)	0 (0–0.05)	0 (0–0.1)	0.196
DBCVA LE	0 (0–0)	0 (0–0.05)	0 (0–0.1)	0.048
Sph Eq RE	0.45 ± 2.41	0.37 ± 1.52	0.14 ± 2.13	0.143
Sph eq LE	0.71 ± 2.12	0.14 ± 1.50	0.10 ± 1.78	0.457
IOP RE	12.00 ± 2.38	12.51 ± 2.40	14.16 ± 1.88	0.022*
IOP LE	12.00 ± 2.38	12.82 ± 2.34	14.37 ± 2.38	0.038*
Schirmer test ≤ 5 mm worse eye	1 (28.6%)	7 (21.2%)	7 (29.2%)	0.693
BUT < 7’ worse eye	1 (14.3%)	3 (9.1%)	0 (0%)	0.155
Fluorescein stain ≥ 3 worse eye	1 (14.3%)	6 (18.2%)	2 (8.3%)	0.594
Personal history of dry eye or severe symptoms^ϕ^	5 (71.4%)	5 (15.1%)	6 (25.0%)	0.011
Dry eye disease diagnosis (signs and symptoms)	1 (14.3%)	4 (12.1%)	2 (8.3%)	0.867
Ocular pain	0 (0%)	4 (12.1%)	3 (12.5%)	1.000
Blurry vision	2 (28.6%)	17 (51.5%)	11 (45.8%)	0.589
Non-proliferative DR	0 (0%)	3 (9.1%)	7 (29.2%)	0.069

Data shown in mean ± SD, median (Interquartile ranges) or frequency (%). VA: visual acuity; RE: right eye; LE: left eye; DBCVA: distant best-corrected visual acuity; Sph Eq: Spheric equivalent; IOP: intraocular pressure; BUT: break-up time; DR: Diabetic retinopathy; ^ϕ^Dryness or irritation; * (One-way ANOVA, between severe and critical).

Concerning blurry vision, 30 out of 64 (46.8%) presented this complaint at the moment of the eye examination, but only 20 (31.2%) reported that it appeared or worsened simultaneously with COVID-19. Of the seven patients (10.9%) who reported ocular pain at the moment of eye examination, all of them had this complaint previously to the COVID-19, and only one reported worsening pain in the acute phase.

There were no findings related to anterior or posterior segment uveitis. Concerning the posterior pole findings associated with previous comorbidities, the three main findings were: 10 (15.6%) were diagnosed as non-proliferative diabetic retinopathy, 11 (17.2%) presented increased retinal vascular tortuosity and 3 (4.7%) had glaucoma diagnosis. Considering that there were 19 patients with diabetes mellitus, the frequency of non-proliferative diabetic retinopathy in this sample was (10/19) 52.7%.

**Figure 1:**
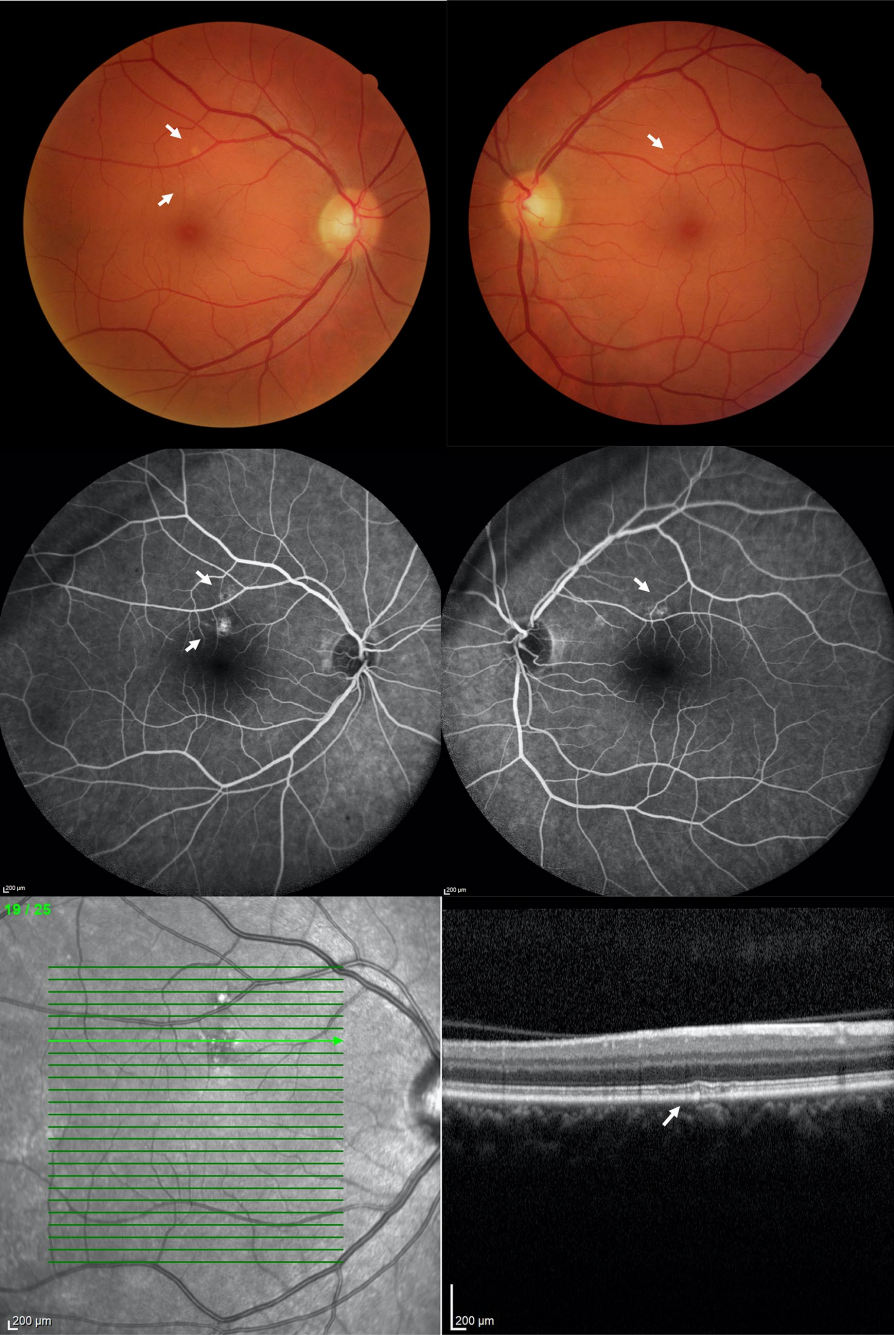
Ocular Fundus multimodal imaging of a 48-year-old man (critical case) 128 days after first symptoms of COVID-19. Color fundus pictures of both eyes showing white-yellowish dots (arrows). Midphase fluorescein angiography pictures of the RE (middle left) and LE (middle right) showing transmission hyperfluorescence in the retina lesions 195 days after first symptoms of COVID-19. Optical coherence tomography (OCT) of the right eye shows hyporreflectivity in the retinal pigment epithelium and ellipsoid layers, and discontinuation of photoreceptors’ outer segments (arrow). (Fig. 1 is composed of six individual photographs, using the Microsoft^®^ PowerPoint for Mac software, version 16.47 (21,031,401), author IFC).

Two out of 64 patients (both from the critical group) presented with a white-yellowish lesion in the posterior pole, one in both eyes (Fig. 1) and the other in the RE. These lesions showed hyporreflectivity of the retinal pigment epithelium and ellipsoid layers and discontinuation of photoreceptors’ outer segments in SD-OCT, and transmission hyperfluorescence in fluorescein angiography. The remaining patients had unremarkable exams.

## Discussion

This study evaluated the ocular findings of patients who recovered from COVID-19 with a mean time of 82 ± 36.4 days after the onset of the disease's first symptoms.

Concerning visual acuity, only two eyes of two patients presented DBCVA > 0.5 logMAR, and both had cataracts diagnosis before COVID-19 onset; indeed, it is one of the leading causes of visual impairment at this age range (50 years)^21^.

Regarding refractive errors, as the Pearson correlation between RE and LE was 0.85, we used only the RE for the refractive error data analysis. The frequency (n = 63) of myopia (sph eq < -0.50D) and hyperopia (sph eq >  + 0.50D) was respectively 31.7% and 41.2% and seems not to be different when compared to prevalence data on refractive errors. In a Brazilian study, the myopia’s prevalence varied from 10 to 35% (ages ranging from 30 to 59 years old), and hyperopia varied from 30 to 60%^22^. Besides, according to a meta-analysis on refractive errors^23^, the estimated pool prevalence of myopia and hyperopia for adults in South America is respectively 22 and 37.2%.

The absence of previous signs of uveitis in the anterior and posterior segments, associated with mean tonometry varying from 12.00 to 14.37 mmHg, reinforces a few ocular inflammation cases associated with COVID-19 that only occasionally leave eye sequelae^24,25^. The mild but statistically significant increase in the intraocular pressures observed between severe and critical cases might be associated with the systemic use of corticosteroids in a large portion of patients (48.3% used it in this sample)^26^.

Regarding data related to dry eye disease and symptoms, Alves et al.^18^ showed a previous overall diagnosis of dry eye in 10.2% of the sample and the presence of severe symptoms in 4.9%. Our data shows a higher previous diagnosis of dry eye or severe symptoms, especially in women (38.7%), which may be due to a more advanced age of the female patients (54.2 ± 14.7 years) in this sample. The higher frequency of personal history of dry eye or severe symptoms in mild-to-moderate cases needs other studies to be clarified due to a small number of patients in the sample.

Our sample showed a higher percentage (46.8%) of patients complaining of blurry vision when compared to a previous study^5^. However, only 31.2% reported that it appeared or worsened simultaneously with the acute phase of COVID-19. Possibly a percentage of this complaint may be related to previous uncorrected refractive errors since presenting visual acuity and DBCVA statistically improved in all the groups, except for the LE in the mild-to-moderate group.

Regarding diabetic retinopathy, its frequency was 52.7% in our sample, which is a higher frequency than the overall prevalence of diabetic retinopathy in a previously diabetic population (34.6%)^27^. Future studies are needed to investigate whether diabetic retinopathy is a predictor for the severity of COVID-19.

The retina white-yellowish dots verified in two of our patients (both critical cases) are similar to the ones described by Zago Filho et al.^28^ Our lesions were mainly concentrated in the outer retina, while in the former study, there were hyperreflective points observed in the vitreous and hyperreflective lesions in the level of inner plexiform and ganglion cell layers, in addition to outer retina changes. The vitreous and inner retina changes may be related to the acute phase of the infection since the reported patient had only 12 days of symptoms, while in the present study, patients were examined 128 and 110 days after the first symptoms. Corroborating the report from Zago Filho, the lesions in our patients were also self-limited, with a good visual prognosis^28^.

Our study presents some limitations. First, from the recruitment perspective, the participants might have been more likely to accept the invitation to join the study if they had ophthalmologic symptoms. Therefore, our data is subjected to selection bias and may overestimate the frequency of some abnormalities found. Second, participants were not evaluated at the acute phase of the disease, so we do not know their previous ophthalmologic status. Third, we did not control our data for climate factors, which may have influenced the results related to dry eye signs and symptoms.

In conclusion, 51.5% of patients surviving the acute phase of COVID-19 were clinically classified as severe and 37.5% as critical. Only subtle retinal changes with a good visual prognosis may be directly related to COVID-19 infection in the medium-term. No sign of uveitis was found. The higher mean IOP in critical cases may be related to disease treatment; other ocular findings, such as diabetic retinopathy, may be associated with the systemic diseases that made those patients more susceptible to COVID-19 clinical manifestations.
